# Development and Pre-Clinical Evaluation of Recombinant Human Myelin Basic Protein Nano Therapeutic Vaccine in Experimental Autoimmune Encephalomyelitis Mice Animal Model

**DOI:** 10.1038/srep46468

**Published:** 2017-04-20

**Authors:** Medhat A. Al-Ghobashy, Aliaa N. ElMeshad, Rania M. Abdelsalam, Mohammed M. Nooh, Muhammad Al-Shorbagy, Götz Laible

**Affiliations:** 1 Analytical Chemistry Department of, Faculty of Pharmacy, Cairo University, Cairo, Egypt; 2 Bioanalysis Research Group, Faculty of Pharmacy, Cairo University, Cairo, Egypt; 3 Pharmaceutics and Industrial Pharmacy Department, Faculty of Pharmacy, Cairo University, Cairo, Egypt; 4 Pharmacology & Toxicology Department, Faculty of Pharmacy, Cairo University, Cairo, Egypt; 5 Biochemistry Department, Faculty of Pharmacy, Cairo University, Cairo, Egypt; 6 AgRresearch, Ruakura Research Centre, Hamilton, New Zealand

## Abstract

Recombinant human myelin basic protein (rhMBP) was previously produced in the milk of transgenic cows. Differences in molecular recognition of either hMBP or rhMBP by surface-immobilized anti-hMBP antibodies were demonstrated. This indicated differences in immunological response between rhMBP and hMBP. Here, the activity of free and controlled release rhMBP poly(ε-caprolactone) nanoparticles (NPs), as a therapeutic vaccine against multiple sclerosis (MS) was demonstrated in experimental autoimmune encephalomyelitis (EAE) animal model. Following optimization of nanoformulation, discrete spherical, rough-surfaced rhMBP NPs with high entrapment efficiency and controlled release pattern were obtained. Results indicated that rhMBP was loaded into and electrostatically adsorbed onto the surface of NPs. Subcutaneous administration of free or rhMBP NPs before EAE-induction reduced the average behavioral score in EAE mice and showed only mild histological alterations and preservation of myelin sheath, with rhMBP NPs showing increased protection. Moreover, analysis of inflammatory cytokines (IFN-γ and IL-10) in mice brains revealed that pretreatment with free or rhMBP NPs significantly protected against induced inflammation. In conclusion: i) rhMBP ameliorated EAE symptoms in EAE animal model, ii) nanoformulation significantly enhanced efficacy of rhMBP as a therapeutic vaccine and iii) clinical investigations are required to demonstrate the activity of rhMBP NPs as a therapeutic vaccine for MS.

Multiple sclerosis (MS) is an autoimmune neurodegenerative disease characterized by inflammatory lesions and demyelination in the central nervous system (CNS)^1^. Patients with this disease suffer from several disabilities like memory dysfunction, cognitive deficit and movement disorders^2^. Approved drugs for treatment of MS that non-specifically inhibit the immune system are often associated with serious side effects. On the other hand, targeting pathogenic T-cell response offers a better opportunity to treat the disease^3^,^4^. Several peptide-based therapeutics that are able to restore immunological tolerance; termed as “therapeutic vaccines” have been reported and some of them are under clinical trials^4^,^5^,^6^. Although the last decade witnessed major breakthroughs in development of new therapies for MS, a systematic review to evaluate their efficacy revealed widely variable efficacy among currently available therapies^7^. Various therapies are under study in phase II or III clinical trials, and some have quite promising effects on clinical and motor disruptions associated with MS in early phases. Amiloride, high dose erythropoietin, MIS416 (a myeloid-directed microparticle immune response modifier derived from *Propionibacterium acnes*), natalizumab, NeuroVax (T-cell receptor peptide vaccine), siponimod, and epigallocatechin-3-gallate are amongst the most important agents being tested now especially for the progressive form of MS^8^.

Human myelin basic protein (hMBP) is considered as the autoantigen in MS pathogenesis process. Presence of hMBP in the cerebrospinal fluid at levels higher than normal (>4 ng/mL) is a marker of active inflammation and myelin breakdown^9^,^10^. Recent studies revealed that administration of the hMBP or its antigenic fragments to MS patients, reduced the severity of the disease through a tolerance mechanism which involves the occurrence of clonal anergy or leads to a bystander suppression via suppressive cytokines release especially IL-10^3^,^11^. Glatiramar acetate (Copaxone); random copolymer of four amino acids found in hMBP is an approved treatment of MS and is an effective suppressor of experimental autoimmune encephalomyelitis (EAE) in various species^12^.

Due to the complex pattern of posttranslational modifications (PTM) of hMBP, simple recombinant systems were incapable of producing biosimilar protein with equivalent properties to the human counterpart. In previous work, transgenic technology has been employed for the production of recombinant (rhMBP) in milk of transgenic cows as an N-terminal His-tagged fusion protein with a molecular weight of 17.2 kDa^13^,^14^,^15^. Several molecular weight (MW) and charge isoforms were detected^14^. Differences in the binding patterns of either hMBP or rhMBP with anti-hMBP antibodies and milk caseins were demonstrated using surface plasmon resonance (SPR)^13^. Results explained the differences in antigenicity of rhMBP when compared to hMBP and the exclusive association of rhMBP with casein micellar phase of milk through calcium-mediated interaction. The reduced antigenicity of the rhMBP was attributed to differences in PTM pattern and suggested the possible use of rhMBP as a therapeutic vaccine for treatment of MS^13^. This approach has been previously proposed and activity of altered peptides was attributed to either modifying T cell activation and cytokine production through exhibiting antagonist or partial agonist signals^4^, or to inducing Th2 phenotype T-cell population that mediates bystander suppression^16^,^17^.

Pharmaceutical biotechnology involves the use of proteins and peptides as bioactive drugs for treatment of various diseases^18^. However, the hydrophilic nature, complex architecture, poor stability and bioavailability of proteins have always been the main limitations. Formulation into nanoparticles (NPs) has been demonstrated to assist the delivery of biologicals by preserving their stability and molecular structure in addition to controlling the release pattern^19^,^20^. Moreover, using hydrophobic polymer-based NPs allowed the delivery of hydrophilic drugs such as proteins across the blood brain barrier (BBB). This has been employed in treatment of several CNS disorders such as Alzheimer, Parkinson’s disease and MS^21^,^22^. One of the hydrophobic polymers that has been used to deliver hydrophilic compounds across the BBB is poly (ε-caprolactone) (PCL). It is an FDA approved, biocompatible and biodegradable polymer due to the hydrolytically labile ester groups in its main chain^23^. Owing to its slow degradation, it has been also used for the preparation of long term implantable devices^24^,^25^. Unlike polylactic polyglycolic acid copolymer (PLGA), PCL does not generate acidic oligomers (lactic/glycolic acids) during biodegradation. Such oligomers have been previously shown to increase the acidity of microenvironment around the protein that could affect its stability^26^. Previous reports utilized PLGA as carrier for MBP antigens in the induction of antigen-specific T cell tolerance in mice using the EAE model^27^.

In the present study, rhMBP was purified from milk of transgenic cows using a vacuum-driven cation exchanger. The rhMBP was formulated into PCL NPs and assessed as a therapeutic vaccine for protection against EAE symptoms in mice. To achieve this objective, rhMBP-loaded PCL NPs were prepared and characterized in terms of: entrapment efficiency (EE%), size, morphology, charge, and release pattern. The behavioral, histopathological and biochemical assessment of the efficacy of rhMBP PCL NPs in the EAE animal model were undertaken. Results were demonstrated along with those obtained following the administration of free rhMBP in order to provide a proof of concept for the possible activity of rhMBP. Differences in activity between the immediate release and sustained release NPs formulations were investigated and correlated with the experimental findings.

## Results and Discussion

### Downstream purification and characterization of rhMBP

**Figure 1:**
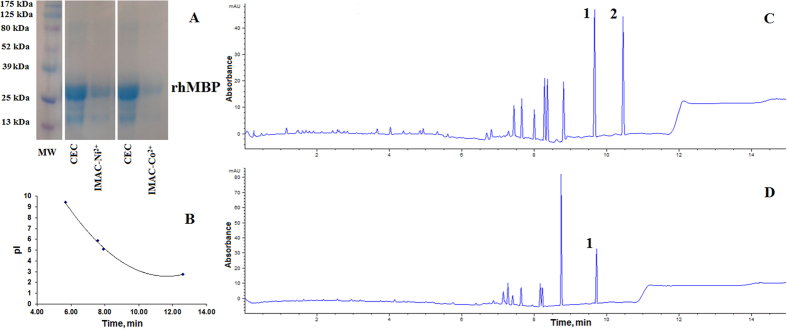
(**A**) SDS-PAGE results showing the relative purity of rhMBP obtained using cation exchange chromatography (CEC) followed by IMAC Co^2+^ when compared to IMAC Ni^2+^. Capillary isoelectric focusing electrophoregrams showing the protein composition of transgenic milk fractions obtained after the cation exchange direct capture step (**B**) and further purification using IMAC-Co^2+^ (**C**). A plot of pI vs migration time of protein markers (**D**). (1: β-casein, 2: αs-casein).

Biopharmaceuticals have an inherently complex structure that is crucial for their activity. This poses additional challenges and constraints to their formulation and assessment^28^. Here, the downstream purification of rhMBP was carried out as previously described^14^ but using a vacuum-driven approach^15^. Successful implementation of vacuum-driven downstream purification obviated the need for an expensive protein chromatography system. A strong cation exchanger resin was used for direct capture of rhMBP from milk of transgenic cows. Further purification of the His-tagged rhMBP was achieved using immobilized metal affinity chromatography (IMAC). Gel electrophoresis results revealed that IMAC-Co^2+^ was superior to IMAC-Ni^2+^ with respect to the purity of the obtained protein (Fig. 1A) as will be discussed.

The identity and concentration of rhMBP in chromatographic fractions were determined using dot blot technique as previously described^14^. The purity and physicochemical properties of rhMBP were further investigated using capillary isoelectric focusing (CIEF) technique^29^. Results of analysis of chromatographic fractions (Fig. 1B and C) confirmed the purity of rhMBP in IMAC fractions and the presence of several isoforms of rhMBP with pI~5 − 7 (Fig. 1D) that is much lower than the calculated value for rhMBP (pI 10.5) and higher than those of milk caseins (Fig. 1B and C). This observation was in agreement with our hypothesis about the effect of the PTM on the physicochemical characteristics of the rhMBP^13^,^14^. Throughout this study an orthogonal testing protocol composed of gel electrophoresis, Bradford protein assay and CIEF were employed to ensure the integrity of rhMBP while dot blot technique was used for determination of rhMBP concentration in chromatographic fractions.

### Nanoformulation of rhMBP

Nine formulations were initially prepared (Table 1) using single emulsification-solvent evaporation method to prepare protein loaded NP^30^. The commercially available human serum albumin (HSA) was chosen as a model protein to optimize the method of preparation of NPs. HSA is a water-soluble protein of a molecular weight of 66.5 kDa and pI 4.7^31^; that is quite close to the practically determined isoelectric point of rhMBP isoforms (pI 5–7). Protein concentration was determined using Bradford protein assay throughout this study^32^. All prepared formulations were evaluated with respect to Entrapment Efficiency (EE%), particle size (Z-average) and surface charge (Zeta potential). The formula with optimum performance was then used to prepare rhMBP NPs.

### Entrapment efficiency (EE%)

Results showed that NPs were able to entrap HSA in the range 22.0 ± 0.4 (**F5**) to 49.9 ± 0.9% (**F7**) as shown in Table 2. The considerably low value of EE% could be ascribed to the hydrophilicity and bulkiness of HSA protein molecules. These results were also in agreement to the previous reports where EE% of bovine serum albumin in modified PCL NPs ranged from 16.7 to 49.5%^30^.

### Particle size

Results showed that all NPs were in the nanorange (Table 2) where the size of NPs enclosing HSA ranged from 208 ± 12 (**F8**) to 596 ± 25 nm (**F6**) with polydispersity index (PDI) ranging from 0.147 (**F2**) to 0.315 (**F7**). Being a protein of relatively high molecular weight, HSA produced NPs of relatively larger size when compared to those obtained with small molecular weight pharmaceuticals. This was partly attributed to the hydrophilic nature of the protein which increased the swelling capacity of the NPs^33^. Other factors affecting the size of NPs were the amount and MW of the stabilizer and the amount of the polymer as shown in Table 2 and further outlined below.

### Zeta potential

Zeta potential is the value of charge conferred to NPs and is considered a measure of physical stability of NPs against aggregation^34^. Zeta potential was determined at neutral pH and the results showed that all NPs acquired a negative charge as shown in Table 2. The zeta potential values of HSA NPs ranged from −5.4 ± 0.1 (**F6**) to −22.6 ± 0.1 mV (**F9**). It was previously reported that NPs bearing zeta potential values ranging from −15 to −30 mV were physically stable^35^, thus, it was concluded that the NPs prepared in this study and bearing zeta potential within this range were well-stabilized. The net negative charge was due to both: the ionized carboxyl groups of PCL and the adsorption of polyvinyl acetate, a polymer of multiples of two monomer units: vinyl alcohol and vinyl acetate; with the hydrophobic vinyl acetate part serving as an anchor on the NPs surface^36^. Variation in the MW of PVA and the amount of PCL had a significant effect on the charge of NPs as further discussed below.

#### Effect of solvent type

Initially, two solvents for PCL polymer were trialed; acetone (**F1**) and dichloromethane (DCM, **F2**) respectively as shown in Table 1. Precipitation occurred in case of acetone which was attributed to protein precipitation^37^. On the other hand, DCM is immiscible with water and has very high vapor pressure. Upon mixing with aqueous protein solution, it rapidly diffuses and evaporates resulting in fast formation of NPs enclosing protein molecules^26^. Thus, DCM was used as the solvent thereafter. For all investigated NP formulations, successful emulsification and formation of NPs occurred upon addition of the polymer solution in DCM to the aqueous protein solution followed by evaporation of the organic solvent (Table 2).

#### Effect of PVA volume

Decreasing the volume of 2% PVA from 50 (**F2**) to 10 mL (**F3**) did not affect significantly the characteristics of NPs (EE%, size and charge) as shown in Table 2. Thus the small volume of PVA was used during NPs formulation afterwards to ease the separation of NPs from the free protein.

#### Effect of PVA amount

Increasing PVA from 2 (**F3**) to 4% w/v (**F4**) significantly increased the particle size of NPs from 520 ± 4 to 576 ± 6 nm (Table 2). During NPs formation using solvent evaporation method, PVA molecules are arranged on the droplet interface between oil and water phases. This is necessary to lower the interfacial tension between the two phases helping in emulsification of oil droplets in the aqueous phase and serving as a stabilizer for the formed droplets. Increasing PVA concentration augmented the number of PVA molecules anchored on NPs surface leading to increased particle size. Throughout formation of PLGA NPs using modified spontaneous emulsification solvent diffusion method, Murakami *et al*.^38^ reported that there was a critical amount of PVA needed to stabilize the oil nanodroplets; the smaller the droplets, the larger the surface area and consequently greater amounts of surfactant would be needed. Below this critical amount, oil droplets would coalesce and the emulsion would be unstable; and above it, increased viscosity of PVA would trigger PLGA precipitation around the agitator during formulation^38^. Herein, 2% PVA was sufficient to form a stabilized emulsion with an acceptable low viscosity and consequently smaller particle size; thus, it was chosen for formulating rhMBP. Moreover, it was previously reported that using high amounts of PVA produced NPs with higher amount of residual PVA that had relatively lower cellular uptake^39^.

#### Effect of PVA MW

Changing PVA MW from 31 (**F3**) to 6 (**F5**) and to 88 kDa (**F6**) resulted in an increase in the NPs size from 520 ± 4 to 546 ± 18 and to 596 ± 25 nm, respectively (Table 2). It could be concluded that at 2% w/v concentration, the optimum MW of PVA to achieve a stable emulsion, with suitable viscosity during NPs formation is 31 kDa. Below this (MW 6 kDa), the viscosity was too low, so PVA molecules adsorbed on oil droplets were not sufficient to stabilize the surface and agglomeration of NPs occurred leading to formation of larger NPs. Above it (MW 88 kDa), PVA solution was too viscous to produce a stable emulsion leading to agglomeration and formation of NPs of larger size.

Results revealed an inverse relationship between the MW of PVA and the zeta potential values acquired by the NPs. Zeta potential of NPs prepared by PVA of MW 88, 31 and 6 kDa were: −5.4 ± 0.1 (**F6**), −14.8 ± 0.4 (**F3**) and −21.3 ± 0.1 mV (**F5**), respectively. The stabilization of NPs by PVA is accomplished by steric repulsion due to stabilizer adsorption at NPs surface rather than through electrostatic repulsion. Increasing the surfactant MW led to greater screening of the charges of NPs contributed by PCL. The same effect was achieved by increasing the concentration of PVA used as a surfactant for PLGA; where Sahoo *et al*.^39^ reported that initially PLGA NPs possessed high negative zeta potential (−45 mV) due to the presence of uncapped carboxyl groups at the polymer surface. Adding increasing concentrations of PVA from 0.5 to 5% decreased the zeta potential due to the shielding effect of surface charge of PLGA NPs^39^.

#### Effect of protein amount

Increasing the amount of HSA used to load the NPs from 50 (**F3**) to 100 mg (**F7**), led to a significant 2-fold increase in EE% from 27.3 ± 0.4 to 49.9 ± 0.9%, respectively (Table 2). This was an expected result, as increasing the amount of the protein in the solution before NP solidification has been reported to increase the EE%^40^.

#### Effect of polymer amount

Increasing the amount of polymer from 100 (**F3**) to 200 mg (**F8**) and to 400 mg (**F9**) increased EE% significantly from 27.3 ± 0.4 to 32.5 ± 1.0 and to 35.8 ± 0.9%, respectively (Table 2). This could be explained on the basis of formation of more PCL NPs to entrap more protein. Increasing polymer amount resulted also in a decrease in the time required for polymer precipitation. Similar results were obtained by Sharma *et al*.^41^, where increased EE% of paclitaxel occurred by increasing the amount of PLGA from 10 to 15 and 20%.

Results indicated also that increasing the polymer concentration led to a significant decrease in particle size of NPs from 520 ± 4 (**F3**) to 208 ± 12 (**F8**) and to 388 ± 13 nm (**F9**), respectively. PCL is a very hydrophobic polymer and by increasing its concentration, the viscosity of the organic phase increases, which accordingly decreases the diffusion of the oil droplets through the aqueous medium during the emulsification step. By keeping the homogenizer speed constant, the lower diffusion leads to smaller distance travelled by the oil through the aqueous medium and thus resulting in formation of NPs of smaller particle size (Table 2). There was a direct relationship between the charge on the NPs and the amount of polymer used. Increasing the amount of PCL forming the NPs resulted in an increase in the zeta potential of NPs from −14.8 ± 0.4 (F3) to −16.6 ± 0.4 (F8) and −22.6 ± 0.1 mV (F9), respectively. Only the increase of PCL from 100 to 400 mg was significant. The negative charge of NPs was due to the ionized carboxyl groups of PCL^36^. Increasing the amount of PCL subsequently expanded the number of carboxyl groups leading to a higher zeta potential.

### Morphology

**Figure 2:**
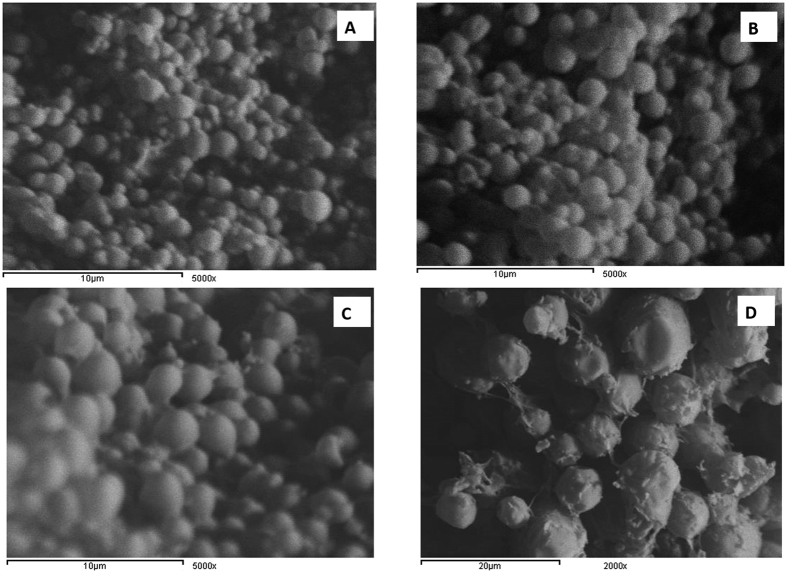
SEM photomicrographs of PCL NPs: (**A**) F3, HSA (scale bar = 10 μm, ×5000); (**B**) F8, HSA (scale bar = 10 μm, ×5000); (**C**) F9, HSA (scale bar = 10 μm, ×5000); and (**D**) rhMBP (scale bar = 20 μm, ×2000).

Figure 2(A–C) displays the scanning electron microscope (SEM) photomicrographs of NPs enclosing HSA (F3, F8 & F9, respectively) that exhibited discrete, spherical, smooth and non-porous surfaces. Moreover, the SEM photomicrographs confirmed the particle size determined by our dynamic light scattering measurements.

### *In vitro* release study

**Figure 3:**
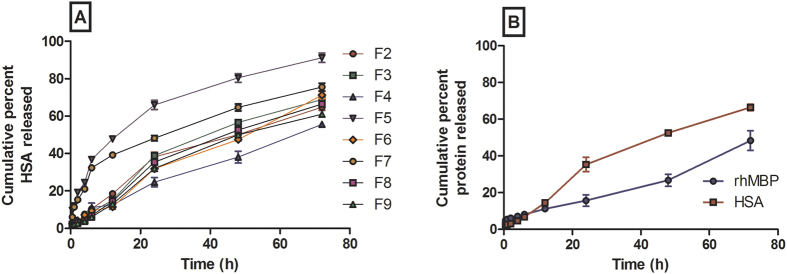
*In vitro* release profile of: (**A**) HSA protein from different NPs and (**B**) different proteins (HSA and rhMBP) from NPs respectively in PBS (pH 7.4) (*n *=* 3*).

The *in vitro* release study (Fig. 3A) showed that HSA was released from NPs in a biphasic pattern. The first stage showed an initial burst release, whereas the second stage exhibited a slower release profile. The biphasic release pattern of HSA from PCL NPs could be explained as follows: the first stage of initial burst release occurred because of the immediate release of the small amount of HSA adsorbed on the surface of NPs. The second stage exhibited a slower release profile due to diffusion of HSA from PCL polymer matrix after erosion of NPs. Almost 10% of HSA was released from all NPs during the first 6 h except **F5** & **F7** where nearly 35% of HSA was released after the same time interval. The afore mentioned formulations exhibited fast protein release compared to the other NPs as 91.2 ± 2.5% (**F5**) and 75.6 ± 2.1% (**F7**) of HSA was released after 3 days, respectively. The fast release of HSA from **F5** was ascribed to the use of low MW and fully hydrolyzed grade of PVA as a surfactant in formulation. Being hydrophilic, water-soluble and of low viscosity, fully hydrolyzed grades of PVA adsorbed on NPs surface weakens the resistance of PCL NPs to dissolution medium due to the presence of numerous vinyl alcohol units which have a high capacity for hydrogen bonding^42^. The fast release of HSA from **F7** could be ascribed to the increased drug: polymer ratio and high EE% compared to other NP formulations, where the polymer (which hindered the protein release) decreased resulting in overall increased protein release. By increasing the amount of PCL polymer forming the NPs from 100 (**F3**) to 200 (**F8**) and to 400 mg (**F9**), the dissolution profile of HSA NPs subsequently decreased. This was because PCL had to degrade in the dissolution medium in order to release of HSA. With greater amounts of the polymer, the distance the protein had to travel before being released into the dissolution medium increased.

To understand the mechanism of drug release, we investigated the kinetics of release of HSA obtained from **F8** and tried to fit them into zero order, first order and Higuchi diffusion models^43^. The release of drug from polymeric matrices might follow a combination of different mechanisms^44^, so Korsemeyer–Peppas model was also used to analyze the release kinetics. The release data were fitted to the equation^45^:


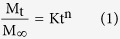


where **M**_**t**_**/M**_**∞**_ represents the drug dissolved fraction at time **t**, **K** is a kinetic constant and **n** is the diffusional exponent. When n < 0.45, this corresponds to a Fickian (case I) diffusion. When 0.45 < n < 0.89, this corresponds to an anomalous (non-Fickian) transport (where release is controlled by a combination of diffusion and polymer relaxation). If n = 0.89, this corresponds to a zero order (case II) transport (where the drug release rate is independent of time and involves polymer relaxation), and n > 0.89 to a super case II transport^46^. However, this equation is valid only for the early stages (≤60%) of drug release^47^.

Results showed that the release of HSA from **F8** followed Higuchi diffusion model. Further analysis of release data of F8 by fitting it into Korsemeyer–Peppas model demonstrated that n-value was 0.762. Since n-value was in the range 0.45 < n < 0.89, thus the release of the HSA was anomalous. This meant that the release of HSA protein from PCL NPs was a combination of diffusion and polymer relaxation (swelling of the PCL polymer followed by erosion).

From the results of the above characterization tests, it was concluded that (**F8**) was the optimum NP formulation in terms of EE%, size, charge and *in vitro* release; thus the method used to formulate (**F8**) was adopted to prepare NP enclosing the rhMBP.

### rhMBP formulation

In case of rhMBP, the EE% was surprisingly high, 99.9 ± 1.7% compared to only 32.5 ± 1.0% in case of HSA (**F8**) as shown in (Table 2). Such significant difference could be attributed to: i) the smaller MW of rhMBP when compared to HSA, ii) random coil structure of rhMBP when compared to the folded structure of HSA that resulted in exposure of the hydrophobic regions of rhMBP to the medium, thus more interaction with the hydrophobic PCL polymer and iii) the abundance of basic amino acid residues in rhMBP sequence that could lead to electrostatic attraction between the positively charged basic amino acids and the negatively charged PCL NPs. The particle size of rhMBP NPs was 295 ± 15 nm with PDI 0.374 whereas its zeta potential was −18.6 ± 0.7 mV (Table 2). The significantly increased particle size of rhMBP NPs compared to HSA NPs (**F8**) could be ascribed to the amount of rhMBP electrostatically attached to the surface of NPs. This agreed to the SEM results, where the micrograph of rhMBP NPs revealed a rough surface (Fig. 2D) which was ascribed to the free rhMBP protein electrostatically adsorbed onto the surface of NPs. From SEM examination, we can draw the conclusion that a proportion of rhMBP was adsorbed on the surface of PCL NPs whilst the remainder was enclosed within the NPs. This conclusion explains the high EE% and the increased size in case of rhMBP NPs when compared to HSA (**F8**).

*In vitro* release study showed that the rhMBP release from PCL NPs was gradual with burst release of 5.5 ± 0.12% after 1 h (Fig. 3B). This could be ascribed to the amount of protein adsorbed onto the surface and immediately released upon addition of NPs to the dissolution medium. This result was in good agreement with those of EE%, zeta potential and SEM micrographs. The respective amount of rhMBP released after 1, 2 & 3 days were 15.7 ± 3.14, 26.8 ± 3.3, and 48.4 ± 5.3%. Figure 3B clearly compares the release pattern of HSA (**F8**) and rhMBP (**F10**) used to load the same type of NPs. Both of (**F8**) and (**F10**) have the same composition and only differ in the type of protein used to load the NPs. The burst amount of rhMBP released was higher than that of HSA. After 1 h, 5.5 ± 0.12% of rhMBP was released whereas only 2.62 ± 2.22% of HSA was released from (**F8**). This confirms that some of the positively charged amino acid residues of rhMBP were electrostatically adsorbed on the negatively charged PCL NPs. The release of rhMBP followed a slower pattern after 1 h compared to that of HSA indicating the ability of PCL NPs to control and sustain the release of rhMBP entrapped within NPs.

As per FDA guidelines for development and approval of nanoparticle therapeutics for human use, robust methodologies for synthesis, characterization, quality control, and potential scale-up should be considered at the early development phases^48^. This should facilitate translation of laboratory scale nanoformulations into pharmaceutical products^49^. Among the numerous nanoformulations; either approved or in clinical trials, liposomes and polymeric-nanoparticles are the most abundant^50^. Generally, drug loading to liposome and polymeric NPs is achieved either via adsorption to the surface or most commonly entrapment within the NPs^51^,^52^.

In a previous study, myelin antigens were covalently coupled to biodegradable PLGA NPs as an alternative to cellular carriers for the induction of antigen-specific T cell tolerance^27^. Authors in that study claimed wide applicability of their approach for treatment of various diseases by switching out the antigenic epitopes conjugated to the NPs^27^. In this study, rhMBP was entrapped within the polymeric PCL nanoparticles. We aimed to i) control the release of rhMBP in a sustained pattern; according to the polymer biodegradation kinetics, ii) enhance the stability of the rhMBP; previously shown to be susceptible to degradation by various experimental conditions^14^,^15^ and iii) most importantly, deliver rhMBP across the BBB via encapsulation into the hydrophobic PCL NPs.

Indeed, there was a small amount of rhMBP (~5%) adsorbed onto the surface of PCL nanoparticles forming a protein corona; as revealed from SEM micrographs and *in vitro* release pattern. Such small fraction was released from the NPs within the first hour of administration and thus had minimal therapeutic effect when compared to the extended duration of release (~70 h). This assumption was further verified from the release kinetics of rhMBP from NPs that followed zero order kinetics; constant rate, independent on the amount of protein loaded in the NPs. In fact, zero order kinetics is the ultimate goal of all controlled-release drug delivery systems for its supreme control of drug plasma concentration thus improving patient compliance and reducing the frequency of drug administration^43^.

### *In vivo* study

To better understand the structural-morphological diversity of MS, several investigations used experimental autoimmune encephalomyelitis (EAE); the experimental animal model of MS induced by active immunization with myelin antigens^53^. Subcutaneous injection of an emulsion containing an adjuvant and peptides derived from myelin proteins such as myelin oligodendrocyte glycoprotein (MOG), myelin basic protein (MBP) or proteolipid protein (PLP) is carried out with a subsequent activation and expansion of antigen specific T cells. Differentiation into inflammatory CD4+ T-helper (TH) cells then occurs in the spleen before migrating to the CNS. Once in the brain, TH are re-stimulated and the consequent inflammation leads to myelin destruction, astrogliosis, and production of chemotactic cytokines^54^.

For the evaluation of the effect of rhMBP either in its free or NP-formulated forms in this study, EAE was induced using a synthetic peptide derived from MOG. In literature, MOG-induced EAE has attracted increasing attention for the following reasons: i) MOG is easily accessible to antibodies since it is expressed on the myelin surface, ii) MOG-reactive T-cells are readily found in the circulation in MS patients, iii) MOG-derived peptides were shown to reproducibly induce EAE in a variety of mice strains^55^,^56^,^57^ and iv) MOG is considered the most important target of B cells and antibodies in EAE that can produce pathogenic demyelinating autoantibodies^5^,^6^. Moreover, MOG is highly encepahalitogenic and T-cell response in MS is predominantly directed to it rather than to other myelin antigens such as hMBP and PLP^55^,^57^.

The encephalitogenicity of MOG has been characterized in different mice strains and it was shown that MOG has a higher encephalitogenic potential in BALB/c mice^58^,^59^. It was previously shown that BALB/c mice were susceptible to EAE induced by immunization with PLP. Moreover, BALB/c mice revealed features typical of EAE in other strains, including mononuclear cell infiltration, myelin loss, and axonal loss^60^.

### Severity of EAE symptoms in different groups

**Figure 4:**
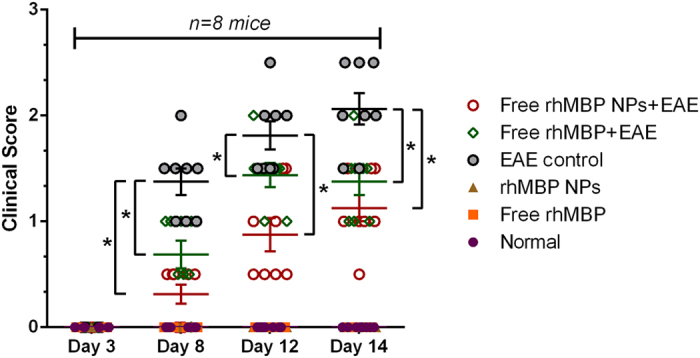
Clinical scores of mice (n = 8) in normal, free rhMBP, rhMBP NPs, EAE control, free rhMBP + EAE, and free rhMBP NPs + EAE groups. Each clinical score on days 3, 8, 12, and 14 was plotted in a scatter chart (mean ± SEM) with statistical significance of scores (*p < 0.05) from EAE control group.

During the experimental study, no mortality was observed in all mice groups under investigation. Onset of EAE disease was observed after 8 days from induction. Mice in the EAE group suffered from the limb defect that reached its top score on day 14 to be 2.1 ± 0.34. Administration of free rhMBP or rhMBP NPs alone did not alter the movement of the mice or show any limb defects, whereas administration before EAE-induction reduced the average behavioral score of mice down to 1.38 ± 0.2 and 1.11 ± 0.18, respectively (Table 3 and Fig. 4).

In the present model, neurologic dysfunctional manifestations observed after immunization with (MOG) 35–55 peptide emulsified in Freund’s are attributed to inflammatory infiltration and demyelination in the peripheral white matter of the spinal cord^61^. Macrophages and CD4+ T cells are the main cell types in the inflammatory infiltrate in the brain^62^. A decline of pro-inflammatory cytokines (TNF-α, IL-17 and IFN-γ) as well as enhancement of the anti-inflammatory cytokine, IL-10, would be predictive of possible amelioration of inflammatory milieu and a subsequent interruption of demyelination process, and could thus explain the observed reduction in neurologic dysfunction scores, indicating a neuroprotective effect of both free rhMBP and rhMBP NPs (Table 3). Accordingly, the effects of rhMBP pretreatments on inflammatory cytokine levels as well as the anti-inflammatory IL-10 were studied.

### Effect of free rhMBP and rhMBP NPs pretreatment on inflammatory cytokines in brains of mice with EAE

Elevation of pro-inflammatory cytokines: TNF- α, IFN-γ and IL-17, (6, 4.9 and 5.3-fold, respectively) was observed in EAE mice brains together with reduction in the anti-inflammatory cytokine IL-10 (3.6 fold) as compared to the normal control (Table 4). Pretreatment with free rhMBP showed significant protection against neuroinflammation. Meanwhile rhMBP NPs revealed better lowering in TNF-α coupled to superior elevation in IL-10 compared to the free rhMBP group.

Previous studies showed that EAE is initiated by autoreactive CD4+ Th_1_ and/or Th_17_ lymphocytes specific for myelin proteins^2^. These cells are activated in the periphery by unknown mechanisms and infiltrate the CNS where they secrete pro-inflammatory cytokines (e.g. IFN-γ, TNF-α, IL-17) to initiate and propagate a pro-inflammatory response leading to demyelination of CNS axons by multiple mechanisms including cytokine-mediated demyelination by TNF-α^63^ and antibody-mediated mechanisms^64^.

*In vitro* and *in vivo* experiments demonstrated that modulation of pro-inflammatory *versus* anti-inflammatory cytokines by protective agents may alter EAE disease course^65^. The neuroprotection afforded by rhMBP in both the free and NPs forms is thought to be by suppressing inflammation and improving cytoprotective mechanisms^66^,^67^. The observed immunomodulatory effect of rhMBP on cytokine production (lowering pro-inflammatory cytokines; IFN-γ and TNFα and IL-17 and augmenting the anti-inflammatory cytokine IL-10) had a beneficial effect on the EAE mouse model and could be expected to have similar beneficial effects on the clinical course of MS^2^,^68^. It’s worth mentioning, when comparing both the free and NPs forms effects, that although they showed comparable reductions in EAE-induced clinical manifestations, the rhMBP NPs showed a preferential superiority in preserving the myelin sheath shown in histopathological sections stained with Luxol fast blue/periodic acid–Schiff’s (LFB/PAS). The latter could be explained in light of the NPs formulation ability to prompt a better reduction in EAE-induced elevation in TNF-α coupled to an increased release of IL-10 reported in the current work, an effect that would reflect less TNF-induced demyelination^63^. The relative superiority of the NPs preparation over the free rhMBP in mediating the currently reported bystander suppression could be linked to the proven ability of the NPs formulation to exhibit a sustained release pattern and thus a relatively better efficacy.

Although administration of native or synthetic myelin derived peptides is considered an attractive approach^3^,^4^,^12^. In the present work rhMBP administered as whole protein showed protection against EAE induced in mice. Such effect can be based on the concept that the hMBP-derived peptides induce a strong regulatory T-cell response via IL-10 producing T cells^69^. This is in line with our results where IL-10 was increased in both groups pretreated with rhMBP (free and encapsulated). Moreover, the possible protective capacity of rhMBP can be due to its anti-inflammatory potential and suppression of pro-inflammatory cytokines; IFN-γ, TNF-α and IL-17 which led to protection against cytokine-mediated demyelination of CNS axon^63^. The slightly altered pattern of PTM in rhMBP could have contributed to such effect without inducing the adverse effects previously noted with altered peptide ligands^4^; produced via substitution of amino acids that recognize T-cell receptors.

It has been reported that using polypeptides longer than 20 amino acids could result in side effects related to IgE-crosslinking on mast cells and basophils^4^. This was not the case with rhMBP which could be attributed to the previously demonstrated weak binding pattern to anti-hMBP antibodies using SPR biosensor^13^. It could be concluded that differences in PTM pattern of rhMBP played a key role and enabled production of a possible promising therapy for MS. Lack of such side effects noted with rhMBP administered as immediate release (Free rhMBP) or sustained release (rhMBP NPs) indicated that the pattern of release is not associated to this finding. Further studies by the authors are being undertaken to foresee the possible therapeutic potential of the rhMBP NPs preparation when given to animals following EAE induction.

### Histopathological findings

**Figure 5:**
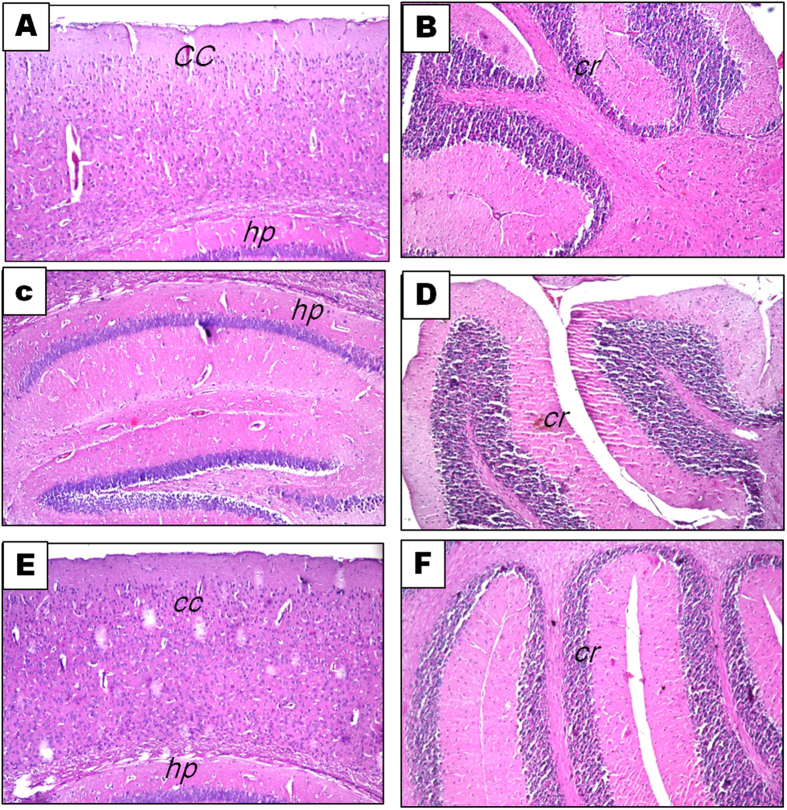
Photomicrographs of brain sections stained with H&E (x16) 14 days post induction. Normal control group: (**A**,**B**), free rhMBP treated control group (**C**,**D**) and rhMBP NPs treated control group (**E**,**F**) showing normal histological structure of cerebral cortex (cc), hippocampus (hp) and cerebellum (cr).

**Figure 6:**
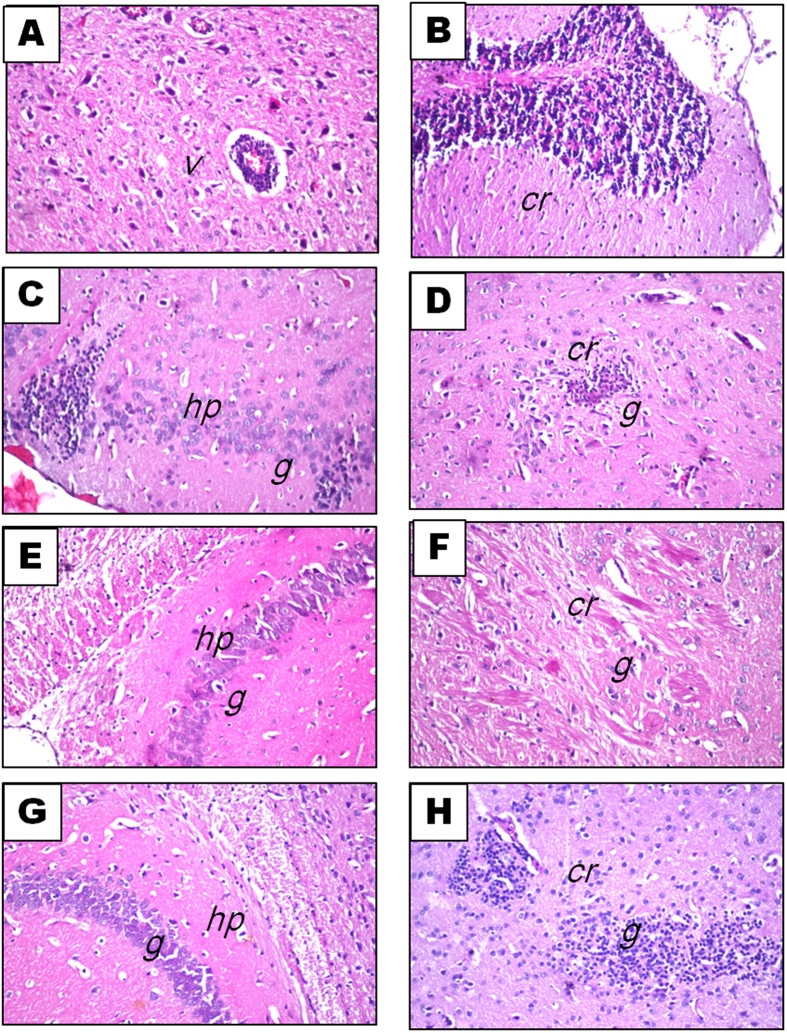
Photomicrographs of brain sections stained with H&E (x16): EAE group showing: (**A**) sclerotic cerebral blood vessels (v), perivascular mononuclear inflammatory infiltrate, average 15/5HPF (**B**) cerebellum (cr) degeneration, inflammatory infiltrate, average 10/5HPF (**C**) hippocampal (hp) neuronal cells degeneration and gliosis (g), inflammatory infiltrate, average 8/5HPF and (**D**) cerebellum (cr) gliosis (g), inflammatory infiltrate, average 10/5HPF. EAE group pretreated with free rhMBP showing: (**E**,**F**) focal gliosis (g) in the cerebellum, inflammatory infiltrate, average 5/5HPF (cr) and hippocampus (hp), inflammatory infiltrate, average 10/5HPF. EAE group pretreated with rhMBP NPs showing: (**G**,**H**) mild focal gliosis, inflammatory infiltrate, average 5/5HPF (g) in the cerebellum (cr) and hippocampus (hp), inflammatory infiltrate, average 4/5HPF.

Behavioral examinations and cytokines assessments were complemented with examination of brain sections stained with hematoxylin and eosin (H&E) as well as scoring of LFB/PAS stained brain sections. H&E stained brain sections of the normal (Fig. 5A and B), free rhMBP treated (Fig. 5C and D) and rhMBP NPs treated (Fig. 5E and F) control mice showed normal histological structure of the meninges, cerebral cortex, cerebrum, cerebellum and medulla oblongata. On the other hand, brains of the EAE group demonstrated severe histopathological alterations (Fig. 6A–D) characterized by focal gliosis in the cerebral cortex, associated with perivascular cuffing surrounding the cerebral sclerotic blood vessels. The cerebellum also showed focal gliosis. On the other hand, brains of mice treated with rhMBP or rhMBP NPs before EAE induction revealed less prominent histopathological changes; focal deep eosinophilic plagues formation in the cerebellum and hippocampal neuronal cells degeneration and gliosis in the rhMBP group (Fig. 6E and F), or focal gliosis in the cerebellum and hippocampus in the rhMBP NPs (Fig. 6G and H).

**Figure 7:**
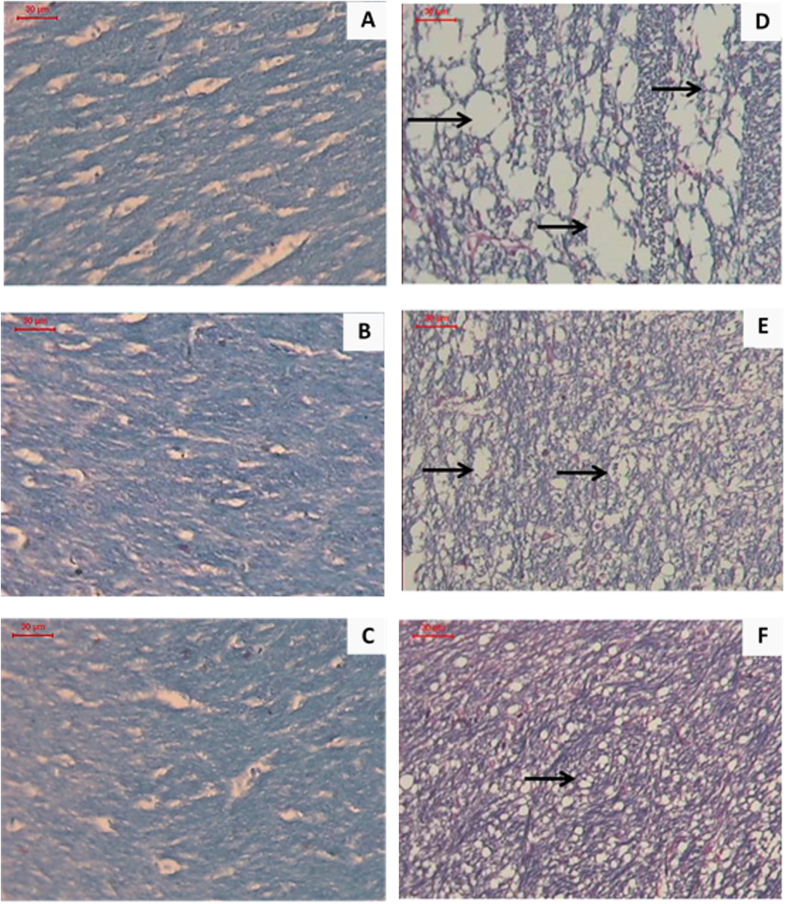
Photomicrographs for LFB/PAS (x200) stained coronal sections of the corpus callosum from (**A**) normal control group (**B**) free rhMBP treated control group (**C**) rhMBP NPs treated control group displaying blue stained tracts in myelinated corpus callosum. (zero score for demyelination) (**D**) EAE group indicating complete demyelination with wide tissue vacuolation and loss of the blue stained fibers. (demyelination score = 3). Arrows point to myelin fragments in the corpus callosum. (**E**) free rhMBP pretreated EAE group showing mild preservation of myelin sheath with focal tissue vacuolation and moderate loss of the blue stained fibers. (demyelination score = 2). Arrows point to myelin fragments in the corpus callosum. (**F**) rhMBP NPs pretreated EAE group indicating better preservation of myelin sheath with minimal tissue vacuolation and mild loss of the blue stained fibers. (demyelination score = 1). Arrow point to myelin fragments in the corpus callosum. The brains were processed 14 days post induction.

LFB/PAS is a commonly used staining procedure to visualize myelin by light microscopy and was used to stain coronal brain sections^70^. For normal (Fig. 7A), free rhMBP treated (Fig. 7B) and rhMBP NPs treated (Fig. 7C) control mice, brain sections displayed blue stained tracts in myelinated corpus callosum (which was scored as zero for demyelination). Mice brains in the EAE group (Fig. 7D) revealed complete demyelination with wide tissue vacuolation and loss of the blue stained fibers (which was scored as three for demyelination). Brains of mice group pretreated with free rhMBP before EAE induction (Fig. 7E) indicated mild preservation of myelin sheath with focal tissue vacuolation and moderate loss of the blue stained fibers (which was scored as two for demyelination). Brains of the group pretreated with rhMBP NPs (Fig. 7F) showed better preservation of myelin sheath (compared to the group pretreated with free rhMBP) with minimal tissue vacuolation and mild loss of the blue stained fibers (which was scored as one for demyelination).

The findings of the present study indicated that rhMBP pretreatment might offer a protective effect against EAE-induced behavioral, histopathological and inflammatory changes in mice. Compared to the free rhMBP, sustained release formulation of rhMBP as NPs showed better protection and ameliorated most of the induced changes. Being innately hydrophobic, PCL NPs offered an improved drug delivery system for rhMBP enabling it to cross the BBB to deliver its drug cargo in the mice brains. Moreover, it is assumed that NPs offered stabilization of rhMBP structure by binding the protein in its active form and lowering the interfacial tension between the surrounding air and water, thereby decreasing the protein denaturation and/or proteolysis^71^.

## Conclusion

In the present study, rhMBP was successfully purified from milk of transgenic cows using a vacuum-driven approach. In order to produce NPs enclosing large amount of rhMBP, with small particle size and high surface charge, less concentration of stabilizer and intermediate concentration of polymer should be used. Being hydrophobic, PCL NPs offered an improved drug delivery system for rhMBP enabling it to cross the BBB to deliver rhMBP in the mice brains. It was demonstrated that rhMBP may have the potential as a vaccine for MS using the EAE model in mice. Pretreatment with rhMBP offered a possible protective effect against EAE-induced behavioral, histopathological and inflammatory changes in mice. Compared to the free rhMBP, formulation of rhMBP as NPs showed a relatively better protection and ameliorated most of the induced changes. Further investigations are necessary to move from preclinical to concrete clinical applications.

## Materials and Methods

### Milk

The entire milk from consecutive afternoon and morning milking was collected and pooled to form a representative one-day sample from transgenic cows. All milk samples used in this study were prepared from defatted, freeze-dried milk powder by dissolving suitable amounts in MilliQ water to 10% w/v concentration.

### Antibodies and chemicals

Standard hMBP (1.0 mg/mL) was purchased from Research Diagnostics (USA) (cat no. RDI-TRK8M79). Rat anti-hMBP monoclonal antibody (cat no. ab7349) and horse radish peroxidase-labeled anti-rat IgG monoclonal antibody (cat no. A5795) were obtained from Abcam (UK) and Sigma (USA), respectively. Nitrocellulose membranes and Bradford reagent were obtained from Bio-Rad (USA). HSA was purchased from MP Biomaterials (France). PCL (average Mn 45000) and PVA with a MW range of 31–50 kDa (98–99% hydrolyzed) were obtained from Sigma, (USA). PVA with MW 6 kDa (fully hydrolyzed) and MW 88 kDa (88% hydrolyzed) were obtained from Merck (Germany) and Acros Organics, Thermo Fisher Scientific (USA), respectively. Pertussis toxins, MOG 35–55 peptide and complete Freund’s adjuvant were obtained form (Sigma, USA). All other chemicals were of analytical grade or higher and were obtained from Sigma, (USA).

### Downstream purification of rhMBP

A vacuum-driven downstream purification was optimized in order to purify the rhMBP from transgenic milk. Chromatographic media were packed in-house into Bond Elut SPE cartridges, 6 mL with two Frits (Agilent Technologies, Germany)^15^. The following chromatographic techniques were implemented in order to achieve high purity in the final preparation: i) cation exchanger chromatography using SP Sepharose FF, CV 3 mL, (GE Healthcare, USA) using 50 m M HEPES buffer (pH 7.0) containing 0.0–1.0 M NaCl and ii) IMAC-Ni or Co^2+^ Sepharose FF, CV 3 mL, (GE Healthcare, USA) using 50 mM HEPES buffer, 0.5 M NaCl (pH 7.0) containing 50–500 mM imidazole as previously described^14^. The identity, purity and integrity of the rhMBP in the final preparation were confirmed using SDS-PAGE followed by western blotting detection with anti-hMBP and anti-His-tag antibodies, as previously shown^14^. The total amount of protein and that of rhMBP were determined using Bradford protein assay^32^ and dot blotting assay using anti-hMBP antibody, respectively^14^,^15^. A previously developed and optimized CIEF assay protocol was employed for the determination of the pI of rhMBP and to verify the purity in the final preparation^29^. Analysis was carried out in dynamically coated fused silica capillaries, total/effective length: 33 cm/24.5 cm × 50 μm I.D., anolyte: 100 mM H_3_PO_4_, catholyte: 20 mM NaOH, focusing and mobilization: 15 kV–35 mbar, temperature: 25 °C, hydrodynamic injection: 950 mbar–2 min and detection: 280 nm.

### Formulation of biodegradable nanoparticles

Single emulsification-solvent evaporation method was utilized to prepare protein loaded NP^30^. PCL polymer was dissolved in 2 mL of either acetone or DCM. The protein and PVA were dissolved in 10 mL of MilliQ water. The polymer solution was then emulsified in the aqueous solution (Homogenizer, WiseMix^TM^ HG15D, Daihan Scientific Co., Ltd, Korea) at 12,000 rpm for 2 min using ice bath. After the formation of stable emulsion, the organic solvent was evaporated by continuous stirring on a magnetic stirrer (WiseStir^TM^, Wisd Lab. Instruments, USA) at 1000 rpm for 2 h till a milky colloidal suspension was obtained. Stirring was continued for another 6 h to evaporate the organic solvent and the resultant dispersion was centrifuged at 15,000 rpm (Megafuge 1.0 R; Heraeus, Germany) for 20 min. The recovered NPs were washed 3 times with MilliQ water and re-centrifuged, followed by freeze-drying (Savant Novalyphe-NL 500) at −45 °C and under vacuum of 7 × 10^−2^ mbar. This method was first employed using HSA as a model protein to optimize the method of preparation and the optimum formula was adopted for preparation of rhMBP PCL NPs. Different NP formulations are listed in Table 1.

### Characterization of NPs

#### EE%

The amount of protein entrapped in NPs was determined by measuring the amount of free protein in the supernatant by Bradford protein assay^32^. The experiment was repeated three times and the following equation was used:


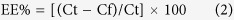


where Ct and Cf are the total and the free protein concentrations, respectively.

#### Particle size and zeta potential

The particle size of different NP formulations was characterized by Zetasizer ZS (Malvern Instruments, UK) based on dynamic light scattering technique at 25 °C after dilution with MilliQ water. Measurements were carried out in triplicate and the mean and standard deviation were deduced. Results were presented as an average diameter of the NP suspension (z-average mean) against percent sample volume The PDI, which is a measure of the width of the size distribution, was also deduced. Zeta potential was measured using a Zetasizer ZS (Malvern Instruments, UK) adopting the principle of electrophoretic mobility under an electric field. Samples were diluted with 1 mM NaCl solution and measured at 25 °C in triplicate.

#### Morphology

The shape of NPs was examined by scanning electron microscopy (SEM, Jeol Electron Probe Microanalyzer, JXA-840A, Japan) at 15 kv. The NPs were coated with a 150 Å layer of gold under vacuum.

#### *In vitro* protein release study

NPs were re-dispersed in phosphate buffer saline (PBS), pH 7.4 and were immersed in shaking water bath (Barloworld Scientific Ltd., UK) at 37 °C and 50 rpm. At specific time periods the tubes were centrifuged at15,000 rpm for 20 min and samples of 1 mL of the medium were withdrawn and same volume of dissolution medium was replaced. The samples were analyzed for protein release by Bradford protein assay^32^. The experiment was repeated three times for each formulation and the mean cumulative percent protein released was calculated according to the following equation:


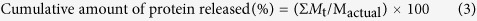


where M_t_ is the amount of protein released at time t, and M_actual_ is the actual amount of protein- loaded onto the NPs. The protein solution obtained after the *in vitro* release study was subjected to SDS-PAGE in order to determine the effect of various experimental conditions of formulation and evaluation of the integrity of the proteins (HSA and rhMPB).

### *In vivo* study

#### Animals

Ninety male Balb/C mice, 20–30 g body weight, were purchased from Theodor Bilharz Research Institute, Giza, Egypt and were left to accommodate for 1 week before the experiment. All animals were allowed free access to diet and tap water. The experimental procedure was conducted in accordance with internationally accepted principles for laboratory animal use and care and was approved by Ethics Committee, Faculty of Pharmacy Cairo University, Cairo, Egypt (PT 1012). The experiment was carried out in accordance with recommendations for the proper use of animals (National Institutes of Health guide for the care and use of Laboratory animals, NIH Publications No. 8023, revised 1978). Animals were divided into 6 groups as follows:

**Group 1 (Normal control):** Animals were given PBS (pH 7.4) as one subcutaneous (sc) injection.

**Group 2 (EAE group):** Animals were given PBS (pH 7.4) as one subcutaneous (sc) injection, and 8 days thereafter EAE was induced.

**Group 3 (free rhMBP treated control group):** Animals were given free rhMBP (100 μg/mouse, sc), as one injection.

**Group 4 (rhMBP NPs treated control group):** Animals were given rhMBP NPs (100 μg/mouse, sc), as one injection.

**Group 5 (free rhMBP pretreated EAE group):** Animals were pretreated by free rhMBP (100 μg/mouse, sc), as one injection 8 days before EAE induction.

**Group 6 (rhMBP NPs pretreated EAE group):** Animals were pretreated by rhMBP NPs (100 μg/mouse, sc), as one injection 8 days before EAE induction.

#### Induction of EAE and scoring of clinical symptoms in mice

Mice were injected subcutaneously with 100 μg MOG35–55 peptide in complete Freund’s adjuvant containing 4 mg/mL Mycobacterium tuberculosis at two sites on the back. Additionally, 300 ng pertussis toxins were given intraperitoneally (ip) on days 0 and 2 after immunization. Animals behavior was observed daily for neurologic dysfunction and on the fourteenth day post EAE induction, mice were scored for neurologic dysfunction according to a 0–5 scale as follows: partial limp tail, 0.5; full limp tail, 1; limp tail and staggering gait, 1.5; paralysis of one hind limb, 2; paralysis of one hind limb and partial paralysis of the other hind limb, 2.5; paralysis of both hind limbs, 3; weakness of the upper limbs, 3.5; weakness of both upper & hind limbs, 4; death, 5^72^,^73^.

After that, animals were killed by decapitation and brains were removed on ice. The brains of three animals in each group were used for histological examination. The rest of the brains were homogenized (Heidolph DIAX 900, Germany) in ice cold saline for measurement of inflammatory markers namely, IL-17, IL-10, IFN-γ and TNF-α using commercially available ELISA kits obtained from Diaclone (France) for IL-17 and RayBiotech (USA) for IL-10, IFN-γ and TNF-α. All biomarkers were determined using the corresponding kits according to manufacturers’ instructions.

### Histological studies

The brains of mice from different groups were transcardially perfused with PBS, followed by phosphate buffered formalin (PBF) pH 7.4, then isolated and fixed in 10% formol saline for 24 h^74^.The fixed brains were dehydrated in ethanol and embedded in paraffin. Sections were cut at 4 μm thicknesses and mounted on glass slides. All histopathologic processing, assessment of specimens and scoring were performed by an experienced histologist blinded to the identity of the sample being examined to avoid any bias.

#### H&E stain

Sections were deparaffinized and stained by H&E stain for examination through the lightmicroscope^75^.

#### LFP/PAS stain

Sections were deparaffinized, rinsed in 100% ethanol and 95% ethanol, and then incubated in a LFB solution (0.01% in 95% ethanol) overnight at 60 °C. Sections were processed in 0.05% lithium carbonate solution, differentiated in 70% ethanol and counterstained with PAS stain.

The demyelination was scored from zero to three in LFB/PAS stained sections, a score of zero referred to a normal myelin status, a score of one is equivalent to demyelination of only one-third of the myelin tract fibers, a score of two is equivalent to demyelination of two thirds of the myelin tract fibers and a score of three refers to complete demyelination^76^.

#### Statistical analysis

Statistical differences were assessed by one-way analysis of variance (ANOVA) and repeated-measures analysis of variance, followed by Tukey–Kramer test for all parameters measured except in comparison of clinical EAE scores which was performed by two-way ANOVA. Graphpad Prism Software v6.01 (La Jolla, CA, USA) was used and differences were considered statistically significant at *P* < 0.05.

## Additional Information

**How to cite this article**: Al-Ghobashy, M. A. *et al*. Development and Pre-Clinical Evaluation of Recombinant Human Myelin Basic Protein Nano Therapeutic Vaccine in Experimental Autoimmune Encephalomyelitis Mice Animal Model. *Sci. Rep.* **7**, 46468; doi: 10.1038/srep46468 (2017).

**Publisher's note:** Springer Nature remains neutral with regard to jurisdictional claims in published maps and institutional affiliations.

## Figures and Tables

**Table 1:** Different NP formulations prepared using HSA.

Formula	Solvent	PCL (mg)	MW PVA (Da)	% PVA (w/v)	Volume of PVA (mL)	Amount of protein (mg)
F1	Acetone	100	31,000	2	50	50
F2	DCM	100	31,000	2	50	50
F3	DCM	100	31,000	2	10	50
F4	DCM	100	31,000	4	10	50
F5	DCM	100	6,000	2	10	50
F6	DCM	100	88,000	2	10	50
F7	DCM	100	31,000	2	10	100
F8	DCM	200	31,000	2	10	50
F9	DCM	400	31,000	2	10	50

PCL: poly(ε-caprolactone); PVA: polyvinyl alcohol; DCM: dichloromethane; HSA: human serum albumin.

**Table 2:** Characterization of different NP formulations.

Formula	EE ± SD (%)	Particle size Z-average± SD(nm)	PDI	Zeta potential± SD (mV)
F1	N/A	N/A	N/A	N/A
F2	25.6 ± 0.4	502 ± 18	0.147	−15.9 ± 7.0
F3	27.3 ± 0.4	520 ± 4	0.255	−14.8 ± 0.4
F4	30.5 ± 0.5	576 ± 6*	0.175	−17.6 ± 7.1
F5	22.0 ± 0.4	546 ± 18	0.148	−21.3 ± 0.1*
F6	25.9 ± 0.6	596 ± 25*	0.257	−5.4 ± 0.1*
F7	49.9 ± 0.9*	508 ± 21	0.315	−17.1 ± 5.6
F8	32.5 ±1.0*	208 ± 12*	0.189	−16.6 ± 0.4
F9	35.8 ±0.9*	388 ± 13*	0.260	−22.6 ± 0.1*
rhMBP	99.9±1.7^@^	295 ± 15^@^	0.374	−18.6 ± 0.7

rhMBP: recombinant human myelin basic protein.

Each value represents mean ± SD (*n* = *3*).

N/A is not applicable.

*Significantly different from the corresponding value of F3 at *P* < 0.05.

^@^Significantly different from the corresponding value of F8 at *P* < 0.05.

**Table 3:** The effect of free rhMBP and rhMBP NPs on clinical scores of EAE in mice 14 days post induction.

Groups	Clinical score
Normal Control	0 ± 0.0
EAE	2.1 ± 0.3^*^
Free rhMBP treated control	0 ± 0.0
rhMBP NPs treated control	0 ± 0.0
Free rhMBP pretreated EAE	1.4 ± 0.2^@^
rhMBP NPs pretreated EAE	1.1 ± 0.2^@^

Statistical analysis was carried out by non-parametric one-way ANOVA followed by Dunn’s multiple comparisons test for comparison of means of different groups.

Each value represents mean ± SE (*n* = *15 mice*).

*Significantly different from normal control group at *P* < 0.05.

^@^Significantly different from EAE-control group at *P* < 0.05.

**Table 4:** The effect of free rhMBP and rhMBP NPs on inflammatory markers in mice brains.

Groups	TNF-α (pg/g wet wt)	IFN-γ (pg/g wet wt)	IL-17 (pg/g wet wt)	IL-10 (pg/g wet wt)
Normal control	13.88 ± 1.20	23.56 ± 1.33	10.44 ± 0.65	53.50 ± 2.15
EAE	84.16 ± 5.31*	115.46 ± 7.20*	55.50 ± 3.01*	14.80 ± 0.92*
Free rhMBP treated control	8.66 ± 0.61^@^	18.46 ± 0.96^@^	7.25 ± 0.32^@^	61.82 ± 2.23^@^
rhMBP NPs treated control	7.22 ± 0.66^@^	15.43 ± 0.82^@^	6.96 ± 0.41^@^	66.60 ± 3.32^@^
Free rhMBP pretreated EAE	38.66 ± 2.11^@^*	53.08 ± 2.23^@^*	31.32 ± 1.25^@^*	31.10 ± 2.02^@^*
rhMBP NPs pretreated EAE	27.48 ± 1.34 ^#@^*	41.20 ± 2.01^@^*	22.34 ± 1.03^@^*	41.12 ± 2.11^#@^

Each value represents mean ± SE (*n *=* 12 mice*).

*Significantly different from normal control group at *P* < 0.05.

^@^Significantly different from EAE group at *P* < 0.05.

^#^Significantly different from free rhMBP pretreated EAE group at *P* < 0.05.
